# The safety and preventive effects of a supraorbital transcutaneous stimulator in Japanese migraine patients

**DOI:** 10.1038/s41598-019-46044-8

**Published:** 2019-07-09

**Authors:** Daisuke Danno, Miho Iigaya, Noboru Imai, Hisaka Igarashi, Takao Takeshima

**Affiliations:** 1 Department of Neurology, Headache Center, Tominaga Hospital, 1-4-48 Minatomachi Naniwa-ku, Osaka-shi, Osaka 556-0017 Japan; 2 Department of Neurology, Kitasato University, Kitasato Institute Hospital, 5-9-1 Shirokane, Minato-ku, Tokyo, 108-8642 Japan; 3 Department of Neurology, Japanese Red Cross Shizuoka Hospital, 8-2 Ohtemachi, Aoi-ku, Shizuoka, 420-0853 Japan; 4 Headache Care Unit, Department of Internal Medicine, Fujitsu Clinic, 4-1-1 Kamikodanaka, Nakahara-ku, Kawasaki, Kanagawa 211-8588 Japan; 5 Division of Neurology, Department of Internal Medicine, Hyogo College of Medicine, 1-1 Mukogawa-cho, Nishinomiya-city, Hyogo 663-8501 Japan

**Keywords:** Phase III trials, Migraine

## Abstract

Cefaly (Cefaly Technology, Seraing, Belgium) is a device that stimulates the bilateral supraorbital nerve transcutaneously. A previous study in Europe proved that Cefaly was an effective and safe device as a preventive therapy for migraine. However, there have been no studies on this device in Asia. We examined the safety and preventive effect of Cefaly for migraine. One-hundred patients were prospectively collected from four headache units in Japan. The inclusion criteria were as follows: 18–75 years of age, migraine with and without aura, and at least 2 attacks per month. A 4-week baseline period was followed by 12-week treatment period. The primary end point was the change from baseline in the number of migraine days at 12 weeks. The secondary end points include the changes of the number of migraine attacks, all headache days, acute medicine consumption days and headache severity. After treatment, a questionnaire survey on the satisfaction of the treatment was administered to the patients. The Friedmann test was used to assess the changes between baseline period and after treatment, and Mann-Whitney U test was used for the comparison of efficacy between chronic migraine and episodic migraine, with and without prophylactic treatment or medication overuse. After 12 weeks of treatment, Cefaly use significantly decreased the number of migraine days (8.16 vs. 6.84; p = 0.0036). Only three subjects (3.0%) dropped out due to the adverse effects; however, no serious adverse events were observed. The compliance of this study was very high at 90.0%. Furthermore, a significant decrease was observed in the number of migraine attacks (5.33 vs. 3.94; p = 0.0002) and the intake of acute antimigraine drugs (8.75 vs. 7.83; p = 0.0166). Cefaly is considered to be a safe and highly tolerable effective device for Japanese patients. Trial registration: This study was retrospectively registered to UMIN-CTR(UMIN000033333) on 10 July 2018.

## Introduction

Migraine is a chronic disorder with a wide range of functional disability, and the prevalence of migraine in Japan is estimated at 8.4%^1^. Prophylactic treatment for migraine is sometimes limited due to side effects^2^.

Recently, studies on the efficacy of neuromodulation as a new strategy for treating primary headaches has been reported, as non-invasive neuromodulation devices are relatively inexpensive and easier to use than more invasive tools. Cefaly (Cefaly Technology, Seraing, Belgium) is a device that stimulates the bilateral supraorbital nerve transcutaneously. In the double-blinded, randomized, sham-controlled PREvention of MIgraine using CEfaly (PREMICE) study in Belgium, the Cefaly device was used on 67 subjects (ages 18 to 65), and the mean number of monthly migraine days decreased significantly from 6.94 to 4.88 (p = 0.023) after 3 months’ treatment^3^. The 50% responder rate was 38.1% in the verum group, which was significantly greater than that of 12.1% in the sham group (p = 0.023). In another survey in Europe, 2,313 triptan users (ages 14 to 87) rented the Cefaly device and were monitored. After an average testing period of 58.2 days, 46.6% of subjects returned the device, while 53.4% were satisfied with the treatment and purchased the device. Only 4.3% of subjects reported adverse events (AEs), including local pain and intolerance of paresthesia; however, they were all minor and fully reversible^4^. This effective and safe device can aid in the development of new strategies for migraine treatment. However, there have been no studies on the application of the Cefaly device in the treatment of patients of Asian ethnicity. A study to evaluate ethnic differences in the tactile detection threshold of 22 men and 22 women in Belgium and in Japan revealed that Japanese women had the lower tactile detection threshold at the cheek skin than Belgian men, with ethnicity having significant effects^5^. In another study to detect ethnic differences in oro-facial somatosensory profiles in 29 Chinese and 29 Danes who were matched for age and gender and healthy, the Chinese subjects showed higher sensitivity in thermal detection, thermal pain, mechanical deep pain and mechanical pain rating parameters in comparison to Danes. From these studies, it is suggested that Caucasians generally demonstrate lower sensitivity than Asians^6^. These findings indicate that the adverse events or efficacy of stimulation differ according to ethnicity, and suggest that the need for an ethnicity-specific evaluation. Hence, we assessed the preventive effect of supraorbital transcutaneous stimulation (STS) using the Cefaly device against frequent episodic migraine and chronic migraine in Japan.

## Methods

Patients were prospectively collected from four headache units in Japan between April and December in 2016. The inclusion criteria were 18 to 75 years of age, migraine with and without aura, including chronic migraine (International Classification of Headache Disorders - third beta edition (ICHD-3beta) 1.2.1, 1.1, or 1.3), and at least 2 attacks and 4 days of migraine days per month^7^. Patients using acute medicine and prophylactic medicine who had not had the prescription changed in the last three months were also included. The exclusion criteria were subjects who had started or changed prophylactic treatments within the last three months, subjects who had received botulinum toxin injection or nerve blocks within the last three months, and subjects with secondary headache except for medication-overuse headache (MOH; ICHD-3beta 8.2). The subjects who had allodynia were also excluded (Table 1).

**Table 1:** Inclusion criteria and exclusion criteria.

**Inclusion criteria**
1) The subjects of 18–75 years old
2) Migraine with or without aura (ICHD-3beta code1.1 or 1.2.1.1) and at least 2 attacks and 4 days of migraine days per month
3) The subjects who are using acute medicine and/or prophylactic medicine without changing the prescription for the last three months
4) The subjects from whom informed consent has been obtained
**Exclusion criteria**
1) Subjects who changed their prophylaxis within the previous 3 months
2) Subjects who received BOTOX or a nerve block within the previous 3 months
3) Subjects with any secondary headache except for MOH
4) Subjects who have severe neurologic and/or psychiatric disorders
5) Subjects who have epilepsy
6) Pregnant or breast-feeding women
7) Subjects who have severe heart, liver, and/or renal dysfunction
8) Subjects who are using opioids
9) Subjects who have allodynia
10) Subjects who have metal and/or electrical device in their body
11) Subjects who are using a cardiac pacemaker and/or implantable cardioverter-defibrillator
12) Subjects whom investigators consider to be ineligible

**Figure 1:**
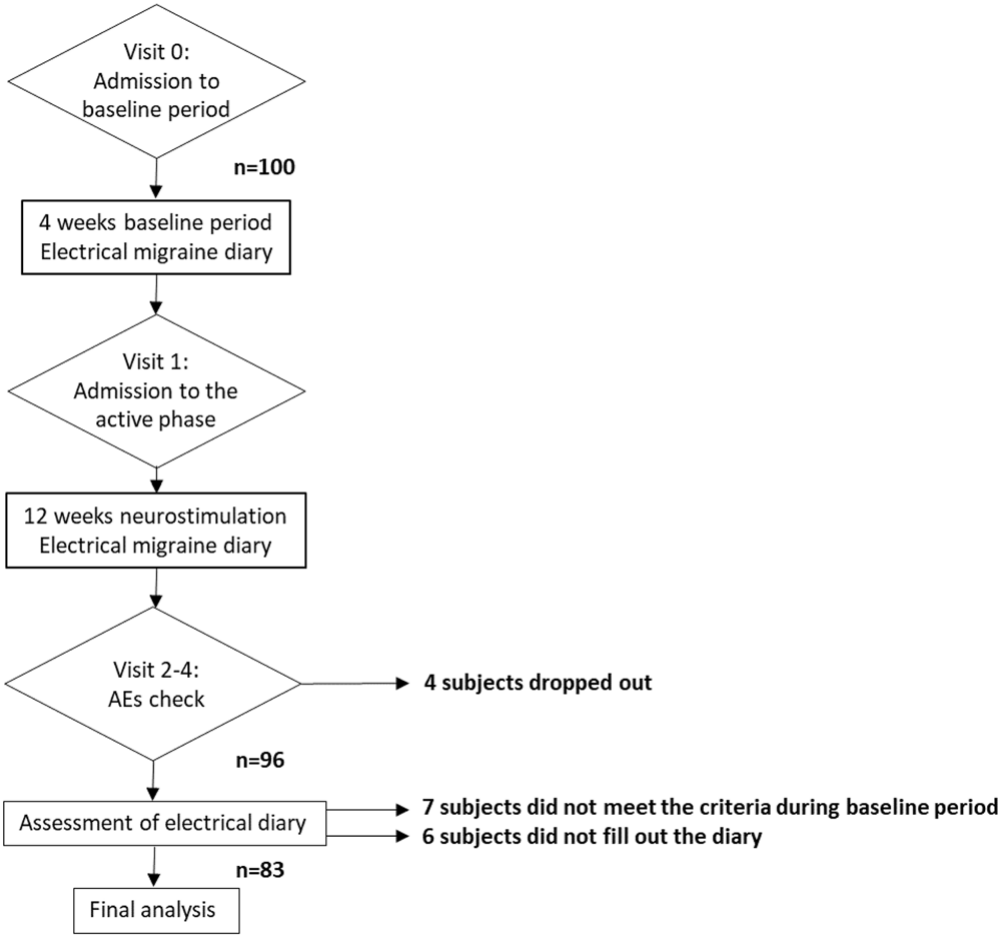
Study design. A 4-week baseline period was followed by 12-week treatment period. A total of 96 subjects completed all 12 weeks of treatment; however, 6 subjects did not fill out the electronic headache diary, and 7 did not meet the inclusion criteria regarding the number of migraine attacks in the baseline period. These 13 subjects were therefore excluded, leaving 83 ultimately included in the analyses.

A 4-week baseline period was followed by 12-week treatment period. The headache and the utilization status of the Cefaly device were recorded separately every day in an electronic headache diary (ZutsuClick; J-MAC SYSTEM, INC., Sapporo, Japan). The primary end point was the change from baseline in the number of migraine days at 12 weeks. The secondary end points include the changes of the number of migraine attacks, all headache days, acute medicine consumption days and headache severity. Headache attacks for which patients used triptan with moderate to severe intensity and with nausea or vomiting were regarded as migraine attacks. After 12 weeks of treatment, all of the collected data was analyzed (Fig. 1).

The Cefaly device generates rectangular waves, with a pulse width of 300 μsec, frequency of 60 Hz, and maximum electric current of 16 mA. All subjects were asked to use the device for 20 minutes every 24 h. After 12 weeks of treatment, a questionnaire survey on the satisfaction of the treatment was administered to the patients. We also analyzed the AEs during the treatment.

The Friedmann test was used to assess the changes between baseline period and after treatment, and Mann-Whitney U test was used for the comparison of efficacy between chronic migraine and episodic migraine, with and without prophylactic treatment or medication overuse. All statistical analyses were performed with the statistical software program EZR^8^.

The study was approved by Tominaga hospital ethics committee, and written informed consent of participance and publication was obtained from all patients. All methods were performed in accordance with the relevant regulations.

### Ethics approval and informed consent

The study was approved by the ethics committee of each participating clinic, and written informed consent of participance and publication was obtained from all patients.

## Results

A total of 100 subjects initially participated in this survey. Three subjects dropped out due to AEs and 1 due to the emergence of allodynia which is one of the exclusion criteria during the treatment period. A total of 96 subjects completed all 12 weeks of treatment; however, 6 subjects did not fill out the electronic headache diary, and 7 did not meet the inclusion criteria regarding the number of migraine attacks in the baseline period. These 13 subjects were therefore excluded, leaving 83 ultimately included in the analyses (Fig. 1). Sixty-eight subjects (81.9%) were women (female:male ratio, 4.53:1). The mean age at entry was 43.6 years (range 27–66 years). The diagnosis was chronic migraine in 23 subjects, 6 of whom had medication overuse headache and 2 who sometimes had migraine with aura. Episodic migraine was noted in 60 subjects, while 55 subjects had migraine without aura, 4 had migraine with and without aura, and only 1 had exclusive migraine with aura.

Throughout the study, a total of seven subjects reported AEs, three of whom dropped out due to sleepiness, headache, the feeling that the stimulus was too strong (one each). The remaining four subjects reported tingling or discomfort at the stimulation site, sleepiness, and fatigue in each case; however, no severe AEs were observed (Table 2). In terms of compliance, instead of 84 stimulation session over 12 weeks, the mean number of sessions was 75.6 days (90.0%).

**Table 2:** Safety and tolerability (numbers of subjects).

Adverse events	Completed the treatment	Dropped out	Total
Sleepiness	1	1	2
Stimulation was too strong		1	1
Tingling at the stimulation site	1		1
Discomfort at the stimulation site	1		1
Fatigue	1		1
Headache		1	1

**Figure 2:**
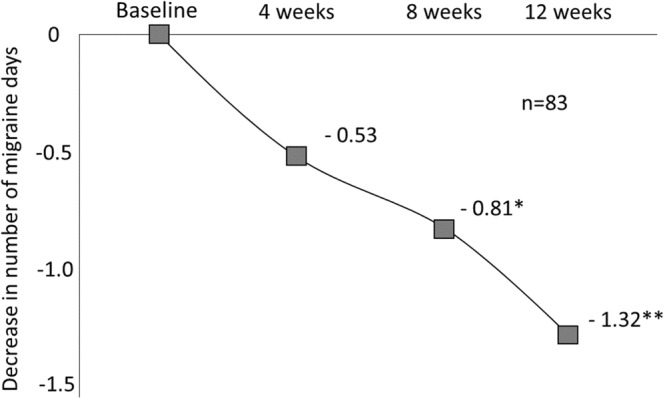
Decrease in number of migraine days (n = 83). The number of migraine days decrease significantly from 8.16 in the baseline period to 6.84 after 12 weeks, with an average reduction of 1.32 days (p = 0.036).

The number of migraine days in the last 4 weeks decrease significantly from 8.16 in the baseline period to 6.84 after 12 weeks, with an average reduction of 1.32 days (p = 0.036) (Fig. 2). The number of migraine attacks in the last 4 weeks decreased significantly from 5.33 in the baseline period to 3.94 after 12 weeks (p = 0.0002). The number of headache days in the last 4 weeks and the consumption of acute medicine also decreased significantly; however, there were no significant changes in the average headache severity (Table 3). The percentage of 50% responder who had at least 50% reduction of migraine days between baseline period and after 12 weeks was 19.3%, and the percentage of 30% responder was 28.9%. We compared the 23 subjects with chronic migraine (CM) and the 60 subjects with episodic migraine (EM), and the average number of migraine days, migraine attacks, all headache days, and acute medicine consumption days in the last 4 weeks were more reduced in the CM subjects than in the EM subjects, although not to a significant degree (Table 4). We also compared the efficacy of the treatment between 53 subjects taking prophylaxis and 30 subjects not taking it and between 6 subjects with medication overuse and 77 subjects without medication overuse, with no specific trends noted between the groups.

**Table 3:** The outcome of this study (n = 83).

	Baseline	4w	8w	12w
**Migraine days/4w**				
Ave	8.16 ± 4.53	7.63 ± 5.01	7.35 ± 4.93	6.84 ± 4.41
*P-value*		*0.118*	*0.0403*	*0.0036*
**Migraine attacks/4w**				
Ave	5.33 ± 3.95	5.04 ± 4.32	4.33 ± 3.19	3.94 ± 2.44
*P-value*		*0.256*	*0.0017*	*0.0002*
**Headache days/4w**				
Ave	11.48 ± 5.70	11.41 ± 6.27	10.4 ± 5.83	9.81 ± 5.66
*P-value*		*0.874*	*0.0201*	*0.0009*
**Acute anti-migraine drug intake/4w**				
Ave	8.75 ± 4.41	8.47 ± 4.91	8.52 ± 5.22	7.83 ± 4.91
*P-value*		*0.487*	*0.569*	*0.0166*
**Severity of headache (NRS)**				
Ave	4.5 ± 1.55	4.08 ± 1.46	4.14 ± 1.61	4.11 ± 1.66
*P-value*		*0.058*	*0.099*	*0.122*

**Table 4:** Comparison of the outcome between chronic migraine and episodic migraine: CM 23 subjects, EM 60 subjects.

	CM	EM
**Migraine days/4w**		
12 weeks after/baseline	0.90 ± 0.70	1.06 ± 1.13
*P-value*	*0.543*	
**Migraine attacks/4w**		
12 weeks after/ baseline	0.75 ± 0.38	0.96 ± 0.70
*P-value*	*0.173*	
**Headache days/4w**		
12 weeks after/ baseline	0.89 ± 0.43	0.99 ± 0.76
*P-value*	*0.552*	
**Acute anti-migraine drug intake/4w**		
12 weeks after/ baseline	0.85 ± 0.65	0.96 ± 0.46
*P-value*	*0.406*	
**Severity of headache (NRS)**		
12 weeks after/ baseline	1.01 ± 0.27	0.97 ± 0.28
*P-value*	*0.555*	

CM: chronic migraine, EM: episodic migraine.

After 12 weeks of treatment, 65.6% of 96 subjects who answered the questionnaire were satisfied, and the reasons for satisfaction were improvement of headache in 44.8% and reduction in the consumption of acute medicine in 35.4%. Furthermore, 87.5% of the subjects used the device at night, and 97.9% reported that installing the electrodes was easy. Regarding whether or not they would purchase the device if it was available, 9.4% said they would ‘be sure to purchase’, and 26.0% would ‘probably purchase’. Of the 63 satisfied with this treatment, 52.4% were willing to purchase the device. Forty-seven subjects experienced headache while using the device, and 40.4% of them reported that the device was effective as an acute treatment.

## Discussion

In this study, we prospectively conducted an open-label trial on the safety and efficacy of STS using the Cefaly device for migraine prevention. This was the first such trial of this device in Asia.

In the post-marketing survey of 2313 Cefaly users in Europe, 99 (4.3%) reported AEs. The most frequently reported AEs were local pain/intolerance to paresthesia in 47 subjects (2.03%), followed by sleepiness/fatigue/insomnia in 19 subjects (0.82%) and headache in 12 subjects (0.52%). A transient local skin allergy was also reported in 2 subjects (0.09%); however, no severe AEs were reported^4^. In our case series, 7 subjects (7.0%) reported AEs, while local unpleasant feelings caused by stimulation and sleepiness/fatigue were the most frequent AEs (both n = 3, 3.0%); 1 subject (1.0%) experienced headache after the session. In a previous study, high-frequency neurostimulation using the device reportedly had a sedative effect in healthy volunteers^9^. This sedative effect might have caused sleepiness/fatigue in our subjects. On the other hand, some of our cases reported that their quality of sleep improved when the Cefaly device was used in the evening. In a population-based study in Korea, the prevalence of insufficient and poor-quality sleep among migraineurs was reported to be significantly higher in comparison to non-migraineurs^10^. It has also been suggested that insomnia may predispose individuals to migraine attacks, and play a role in migraine chronification^11^. In another study, behavioral treatment of comorbid insomnia in individuals with CM was reported to reduce headache frequency^12^. Based on these findings, the moderate sleepiness associated with the use of the Cefaly device might have a positive effect in the treatment of migraine. Unfortunately, we did not collect data on the sleeping patterns or medical comorbidities of the subjects, which is a weakness of the present study. In our study, 3 subjects (3.0%) dropped out due to AEs; however, there were no cases of skin allergy or severe AEs. The details of the AEs were similar to those reported in previous studies. As such, the Cefaly device is thought to be relatively safe for Japanese subjects.

In the PREMICE study, the use of the Cefaly device for 3 months significantly decreased the number of monthly migraine days (6.94 vs. 4.88; p = 0.023), migraine attacks (4.73 vs. 3.55; p = 0.044), headache days (7.78 vs. 5.27; p = 0.041), and intake of acute antimigraine drugs (11.45 vs. 7.25; p = 0.007). However, there were no significant differences in the headache intensity^3^.

Our study employed an open-label design; thus, a simple comparison between the results of the current study and the results of the PREMICE study, which was a double-blind, randomized, and sham-controlled trial, is difficult. However, the outcomes in our study tended to be similar to those of the verum group in the PREMICE study. In particular, the use of the Cefaly device use for 12 weeks significantly decreased the number of monthly migraine days and the intake of acute antimigraine drugs, with no significant differences in the headache intensity noted. The reason for the lower efficacy regarding headache intensity is unclear. In patients experiencing a migraine attack, the metabolic activity of the periaqueductal gray matter (PAG) is reported to be increased on positron emission tomography (PET)^13^, and the PAG is thought to play an important role in initiating the migraine attack^14^. Many of the central nervous system structures including the PAG are known to be activated by electrical stimulating devices^15^, and it is also suggested that the activity levels of the orexin-arcuate-periaqueductal gray matter circuit may change during supraorbital neurostimulation^9^. The possible explanation for the discrepancy in the effectiveness regarding headache frequency and intensity is that the Cefaly device is more effective for raising the threshold of initiation of migraine attack by changing the PAG activity than it is for controlling neurogenic inflammation of the trigemino-vascular system. Further studies are needed to verify whether or not the Cefaly device is effective for reducing headache intensity.

The present study is associated with some limitations, including the lack of a placebo/sham device, and the open-label study design, which means that placebo effects could not be completely excluded. In headache research, the influence of the placebo response must be taken into consideration^16^. The 50% responder rate (reduction of migraine frequency by >50%) was reported to be 55.1% for propranolol and 14.3% for a placebo^17^. Moreover, a sham device had greater effects on self-reported pain than a placebo pill^18^, and the response rates to placebo-acupuncture are reported to reach to 50%^19^. The 50% responder rate in our study was 19.3%, which was lower than that of the PREMICE study (38.1%), and we need to take into consideration the fact that placing an electrical stimulator on the forehead for 20 minutes every day would have a substantial placebo effect. Nevertheless, more than 60% of our subjects were satisfied with the treatment, and more than half of those who were satisfied with the device were willing to purchase it. The reasons for our lower responder rate are unknown, but 76 out of 83 subjects started their treatments from May to August, when are the rainy and typhoon seasons in Japan, respectively. Such changes in weather are common factors exacerbating migraine, and this weather condition might have affected the results^20^. The lower responder rate may also be due to omitted input for the electronic headache diary, especially in the baseline period, due to unfamiliarity with the diary app.

The compliance in the 83 subjects of this study was very high at 90.0% despite the device being time consuming to use (20 minutes daily); indeed, in the previous study, the average compliance was 55.5 days within 3 months (61.7%)^3^. Furthermore, 97.9% of the subjects of our study reported that installing the electrodes was easy. This high compliance in our study was probably due to the high degree of satisfaction and tolerance for the device. Given the present findings, STS with the Cefaly device seems to be a highly tolerable and effective approach to migraine management in Japan.

We compared the improvement rate between the CM and EM groups. No significant difference was noted between the two groups, but the mean improvement rate was higher in the CM group with regard to the number of migraine days, migraine attacks, headache days, and pain-killers being taken. In a previous study in Italy, 23 CM subjects were treated with the Cefaly device for 20 minutes every day, and while 4 subjects dropped out, 8 subjects (34.8%) achieved both a 50% reduction in monthly migraine days and a 50% reduction in monthly medication use after 4 months^21^. The mechanisms of the chronification of migraine are unclear; however, it is suggested that the dysmodulation of the higher central pain pathways, such as the PAG, caused by repeated migraine attacks might be involved^22^. It is also suggested that supraorbital neurostimulation might change the activity levels of the orexin-arcuate-periaqueductal gray matter circuit^9^. In another study using PET, normalization of the fronto-temporal hypometabolism, which is a pivotal area for central pain control, was confirmed by after three months of treatment using the Cefaly device, suggesting for the device to have a central modulatory effect^23^.The reason for the mean improvement rate being higher in the CM group than in the EM group is not clear; however, these central modulatory effects of the device on the central pain matrix structure of CM may have been more effect in CM subjects than in EM ones in our study.

In a previous study, preexisting prophylactic treatment or medication overuse had no influence on whether or not the Cefaly device was effective^21^. In our case series, no significant difference of the treatment response was detected between the subjects with and without prophylaxis or between the subjects with and without medication overuse, which is consistent with the findings of the previous study. Taken together, these findings suggest that migraine prevention using the Cefely device may be promising as an addition to preexisting treatments for migraine, regardless of medication overuse.

The Cefaly device was also effective for treating acute attacks in many subjects in this study. Supraorbital transcutaneous neurostimulation is reported to have sedative effects, and was suggested to be effective as an acute treatment of migraine^9^. However, future prospective studies should be performed to evaluate the efficacy.

Furthermore, in the present study, the treatment period was 12 weeks, and a longer follow up may be necessary to clarify the long-term effects of this device. Nevertheless, this is the first study of Asian subjects under-using prophylaxis, including MOH subjects, thereby providing real-world data and making our findings clinically important.

## Conclusion

This was the first clinical study in Asian subjects to assess the preventive effect of STS using the Cefaly device against frequent episodic migraine and chronic migraine, with our findings suggesting that STS using the Cefaly device in such a setting is a safe and highly tolerable effective treatment. We also showed good results in CM subjects, suggesting that modulating the central pain matrix may have been effective. There were no marked differences in the efficacy between the presence or absence of prophylaxis or medication overuse. Therefore, this device might be a suitable addition to preexisting treatments. Future studies should investigate the long-term migraine-preventive effects and acute effects on migraine attacks of this device.

## Data Availability

The datasets used and/or analyzed during the current study are available from the corresponding author on reasonable request.
